# Treatment strategies to reduce cardiovascular risk in persons with chronic kidney disease and Type 2 diabetes

**DOI:** 10.1111/joim.20050

**Published:** 2024-12-31

**Authors:** Faiez Zannad, Darren K. McGuire, Alberto Ortiz

**Affiliations:** ^1^ Inserm, Centre d'Investigation Clinique Plurithématique 1433, U1116, CHRU de Nancy F‐CRIN INI‐CRCT Université de Lorraine Nancy France; ^2^ Division of Cardiology Department of Internal Medicine The University of Texas Southwestern Medical Center and Parkland Health Dallas USA; ^3^ RICORS2040 Madrid Spain; ^4^ Nephrology and Hypertension Department Hospital IIS‐Fundación Jiménez Díaz UAM Madrid Spain; ^5^ Medicine Department Medicine Faculty, Universidad Autonoma de Madrid Madrid Spain

**Keywords:** cardiovascular risk factors, kidney disease, Type 2 diabetes

## Abstract

Chronic kidney disease (CKD) is a prevalent and progressive condition associated with significant mortality and morbidity. Diabetes is a common cause of CKD, and both diabetes and CKD increase the risk of cardiovascular disease (CVD), the leading cause of death in individuals with CKD. This review will discuss the importance of early detection of CKD and prompt pharmacological intervention to slow CKD progression and delay the development of CVD for improving outcomes.

Early CKD is often asymptomatic, and diagnosis usually requires laboratory testing. The combination of estimated glomerular filtration rate (eGFR) and urine albumin‐to‐creatinine ratio (UACR) measurements is used to diagnose and determine CKD severity. Guidelines recommend at least annual screening for CKD in at‐risk individuals. While eGFR testing rates are consistently high, rates of UACR testing remain low. This results in underdiagnosis and undertreatment of CKD, leaving many individuals at risk of CKD progression and CVD. UACR testing is an actionable component of the CKD definition.

A four‐pillar treatment approach for slowing the progression of diabetic kidney disease is suggested, comprising a renin–angiotensin–system (RAS) inhibitor, a sodium–glucose cotransporter 2 inhibitor, a glucagon‐like peptide 1 receptor agonist, and the nonsteroidal mineralocorticoid receptor antagonist finerenone. The combination of these agents provides a greater cardiorenal risk reduction compared with RAS inhibitors alone.

Early detection of CKD and prompt intervention with guideline‐directed medical therapy are crucial for reducing CVD risk in individuals with CKD and diabetes. Evidence from ongoing studies will advance our understanding of optimal therapy in this population.

## Graphical abstract

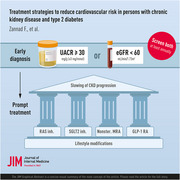

## Introduction

Chronic kidney disease (CKD) is a prevalent and progressive condition affecting over 850 million individuals worldwide [1]. In 2017, the global prevalence of CKD was estimated at 9.1%, and CKD accounts for approximately 1.2 million deaths and >2.5 million people receiving kidney replacement therapy each year [2]. The incidence of CKD is rising faster than most other chronic diseases, which is expected to become the fifth‐leading cause of death globally by 2040 [3].

**Fig. 1:**
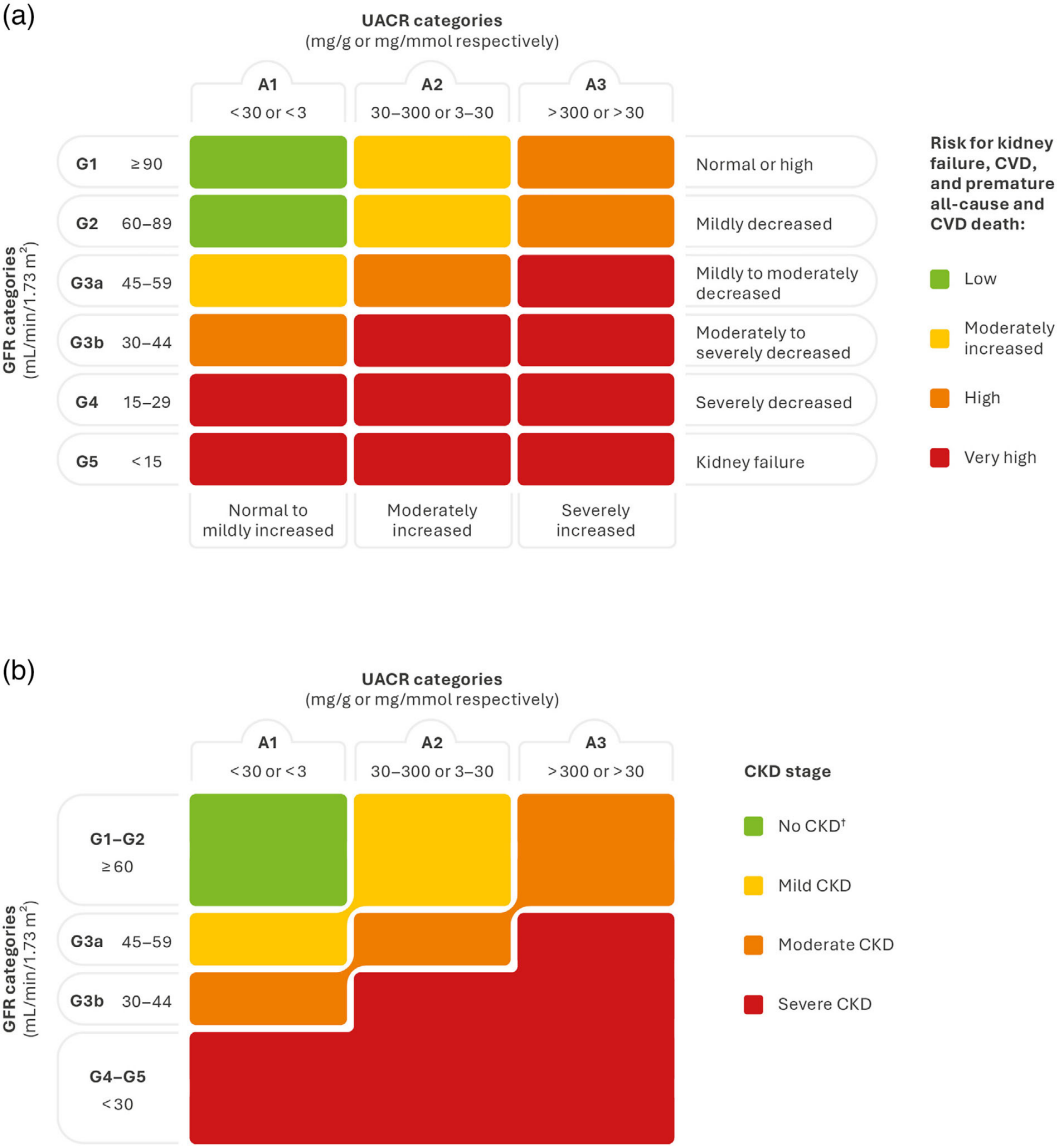
(a) Prognosis and (b) severity of CKD by GFR and albuminuria categories ^†^No CKD if there is no other evidence of kidney damage. A, albuminuria category; CKD, chronic kidney disease; CVD, cardiovascular disease; GFR, glomerular filtration rate; UACR, urine albumin‐to‐creatinine ratio. Source: Panel A adapted from KDIGO 2024 [4] under the terms of the CC BY‐NC‐ND license.

The Kidney Disease Improving Global Outcomes (KDIGO) guidelines define CKD based on the following criteria being present for more than 3 months: estimated glomerular filtration rate (eGFR) <60 mL/min/1.73 m^2^ or markers of kidney damage (one or more structural or functional abnormalities, albuminuria [urine albumin‐to‐creatinine ratio {UACR} ≥30 mg/g {≥3 mg/mmol}], urine sediment abnormalities, persistent hematuria, electrolyte, and other abnormalities due to tubular disorders, abnormalities detected by histology, structural abnormalities detected by imaging, or history of kidney transplantation) [4]. This definition means that CKD may be present even when eGFR is normal. In this regard, an abnormal UACR value alone, if persisting for longer than 3 months, is diagnostic of CKD and should trigger an intervention to decrease CKD‐associated risks. The eGFR and UACR thresholds indicate an increased risk for several adverse health outcomes that are represented in a heatmap where the combination of eGFR and UACR identifies mild, moderate, and severe CKD (Fig. 1) [4, 5, 6, 7]. Among these outcomes, the increased risk of all‐cause and cardiovascular (CV) premature death is not corrected by kidney replacement therapy [8]. The largest loss of life expectancy due to CKD is observed in young adults, with men and women aged 20–24 years who are on dialysis having a shorter life expectancy of 36 and 42 years, respectively, compared with the general population [9].

Diabetes is considered one of the most common causes of CKD, with approximately 30%–40% of people with diabetes developing CKD [10, 11, 12, 13]. Diabetes and CKD are known risk factors for CV disease (CVD), and the presence of both diagnoses has an additive effect on risk prediction for CVD development [12, 14].

Due to the large number of individuals at risk of CVD associated with CKD and diabetes, early detection of CKD and prompt intervention using guideline‐recommended therapies to manage CKD and CVD risk are crucial. However, individuals with CKD still exhibit a dramatically reduced life expectancy, and micro‐ and macroalbuminuria are associated with reduced estimated life expectancy across eGFR categories compared with individuals with normoalbuminuria [15]. Although eGFR is commonly tested among persons at high risk of CKD due to hypertension or diabetes, albuminuria testing is underutilized in these individuals, leading to many at‐risk individuals remaining untreated [16, 17, 18, 19].

The leading cause of death in persons with CKD is CVD rather than kidney failure [14]. There is still an unmet need for additional effective treatments for delaying the development of CVD, slowing CKD progression, and prolonging the survival of people with CKD. This review will discuss the latest developments in the treatment of persons with CKD and diabetes, with a particular focus on managing CVD risk.

### The association between CKD progression and adverse CV outcomes

The prevalence of comorbidities increases with more advanced CKD [20]. Comorbidities may increase CKD disease burden, advance disease progression, lower survival and quality of life, make medication management more complex, and increase healthcare costs [20]. Multimorbidity increases hospitalization rates three to fourfold in people with CKD [21]. Furthermore, comorbidities may lead to reductions in a person's ability to cope with and self‐manage their condition [20].

CKD comes with a pronounced risk for CVD. CKD and CVD may be considered clinical manifestations of the same disorder (chronic CV–kidney disorder); both CVD and CKD share common pathological mechanisms and risk factors [22, 23]. This may present initially as either CKD or CVD, with cardiorenal crosstalk driving further pathological mechanisms of heart and kidney damage [22, 23]. The American College of Cardiology atherosclerotic CVD risk calculator (ASCVD Risk Estimator Plus) may be used to estimate the 10‐year risk of a first atherosclerotic CVD event [24]. The 2021 European Society of Cardiology (ESC) guidelines on CV disease prevention in clinical practice support the use of the Systematic Coronary Risk Estimation (SCORE)2 and SCORE2‐Older Persons algorithms that additionally consider the CVD risk in individual countries [6]. The European Renal Association has also emphasized that SCORE2 algorithms are complemented by the assessment of diabetes and CKD (eGFR/UACR) [5].

Data from the US Renal Data System demonstrate that the prevalence of CVD is 38.4% in individuals aged 18–64 years with CKD compared with 7.1% in those without CKD, and that in persons >65 years, two‐thirds of individuals with CKD have CVD (vs. one‐third without CKD) [25]. Data from a UK cohort study demonstrated that the risk of CVD increases as CKD progresses, with CVD being almost three times as prevalent in CKD stages IV–V compared with stages I–II [20]. Approximately one‐half of individuals with stage IV or V CKD have CVD, and CVD accounts for approximately 40%–50% of all deaths in this group [14]. CV mortality in persons with advanced CKD (stage IV or V) may occur due to multiple etiologies, including fatal atherosclerosis‐related complications such as myocardial infarction (MI) and stroke, heart failure (HF), or fatal arrhythmias [14]. Persons with CKD are also at risk of other CVD risk factors, such as inflammation, oxidative risk, and promoters of vascular calcification [6].

HF is one of the more frequently diagnosed CVDs in individuals with CKD, being present in almost one‐quarter of persons aged >65 years with CKD [25]. In addition, 42%–53% of individuals with HF have comorbid CKD [26]. Following a first hospitalization for HF in persons aged >65 years, the 2‐year survival probability is lower in individuals with CKD compared with those without CKD (0.41 vs. 0.52) [27]. Diabetes frequently coexists with CKD and CVD and is associated with an increased risk of CVD, including HF, and with worse outcomes after CV events [12].

The risk of CVD is raised even in individuals with CKD stage I and II (i.e., normal eGFR, but the presence of UACR >30 mg/g). This was demonstrated in an individual‐participant data meta‐analysis of 27,503,140 individuals across 114 cohorts, which showed that lower eGFR based on creatinine alone, lower eGFR based on creatinine and cystatin C, and more severe UACR were each associated with adverse CV outcomes, including CV mortality, HF, and atrial fibrillation [7]. For eGFR, the associations with outcomes were stronger and more linear for eGFR based on creatinine and cystatin C compared with eGFR based on creatinine alone. Among adults aged ≥65 years, differences in risk reductions (RRs) between eGFR based on creatinine alone and eGFR based on creatinine and cystatin C were observed, suggesting that additional use of cystatin C may improve risk assessment in older populations [7]. For UACR, even mild elevations (UACR of 30–299 mg/g) were associated with increased risk for all outcomes, even in the elderly [7].

Previous meta‐analyses indicated that the risk for CV mortality is elevated in individuals with eGFR <75 mL/min/1.73 m^2^ [28]. A linear relationship between CV mortality and eGFR below this level has been demonstrated, such that CV mortality risk doubled in individuals with eGFR 30–59 mL/min/1.73 m^2^ and tripled in those with eGFR 15–29 mL/min/1.73 m^2^ compared with those with eGFR ≥75 mL/min/1.73 m^2^ [28].

**Fig. 2:**
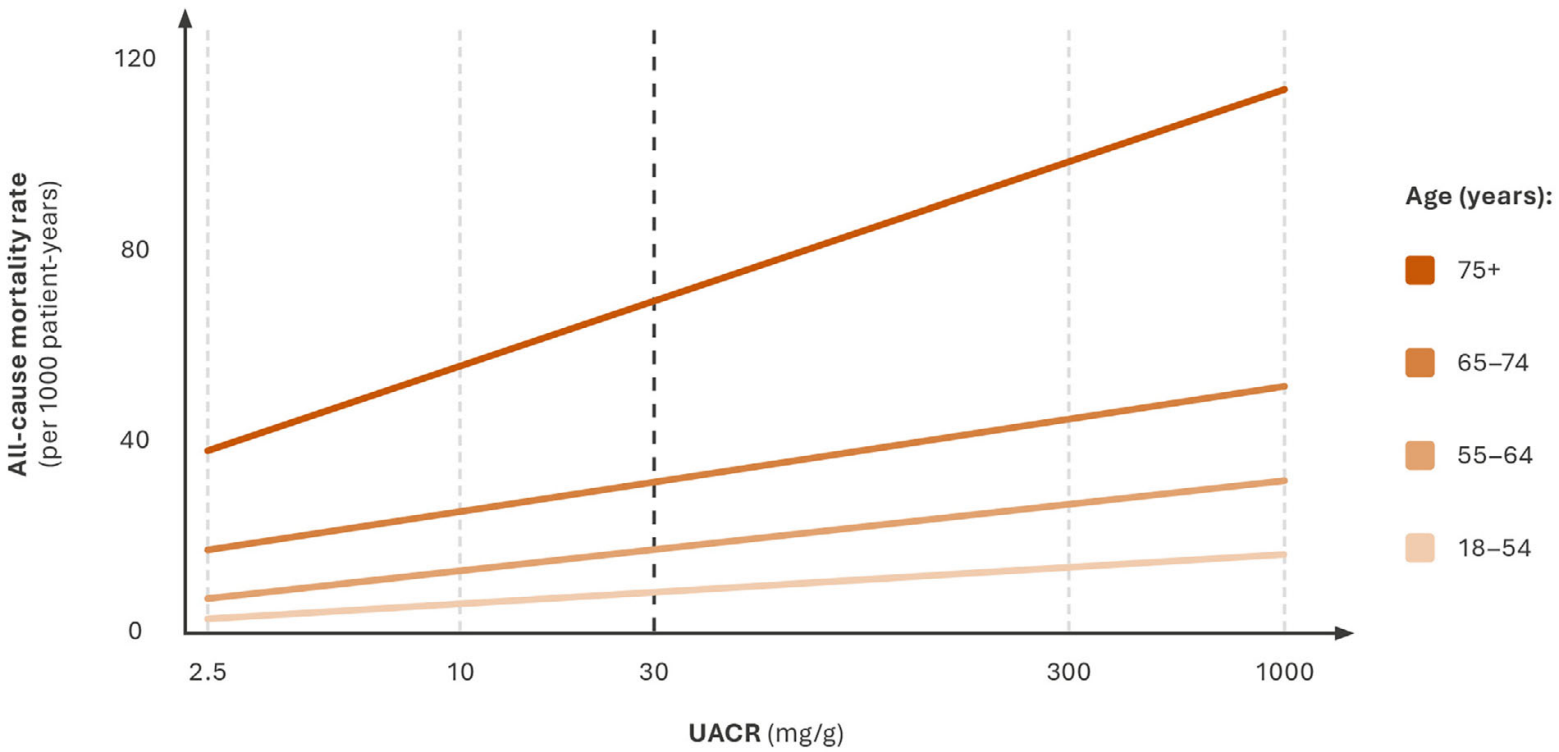
All‐cause mortality rates according to UACR by age category. UACR, urine albumin‐to‐creatinine ratio. Source: Conceptual image derived from Hallan et al. [30].

A linear relationship also exists between UACR and all‐cause and CV mortality within the normal range of UACR, with even mild elevations in UACR (5 to <10 mg/g) associated with increased all‐cause mortality [29]. The association was observed irrespective of CKD risk factors (e.g., CVD, hypertension, and diabetes), and although increased UACR increased the relative mortality risk to a greater extent in younger individuals, the relationship between UACR and all‐cause mortality was still evident in the elderly population [29], in which the largest increase in absolute risk of death associated with high UACR values was observed (Fig. 2) [30].

A previous meta‐analysis, including a total sample size of 693,816 participants, also demonstrated that all‐cause mortality and CV mortality were related to changes in albuminuria. A 43% increase in UACR was associated with an adjusted hazard ratio (HR) of CV mortality of 1.14 (95% confidence interval [CI] 1.06–1.22) [31]. A further meta‐analysis, including 637,315 participants without a history of CVD, reported that UACR detection was significantly associated with improved discrimination of CV outcomes such as coronary heart disease, stroke, or HF over a 5‐year time frame [32]. This was especially evident in participants with diabetes [32], a finding that was in agreement with another study, specifically in individuals with Type 2 diabetes (T2D) without established CVD, which showed an association between UACR and CV events [33].

### Early detection of CKD and comorbidities: the role of UACR screening and albuminuria

Early detection and timely intervention for CKD are critical for slowing disease progression and preventing comorbidities, including CVD. Early kidney disease is often asymptomatic, with few specific symptoms presenting until kidney failure occurs [34]; therefore, early diagnosis usually requires laboratory testing [35].

**Fig. 3:**
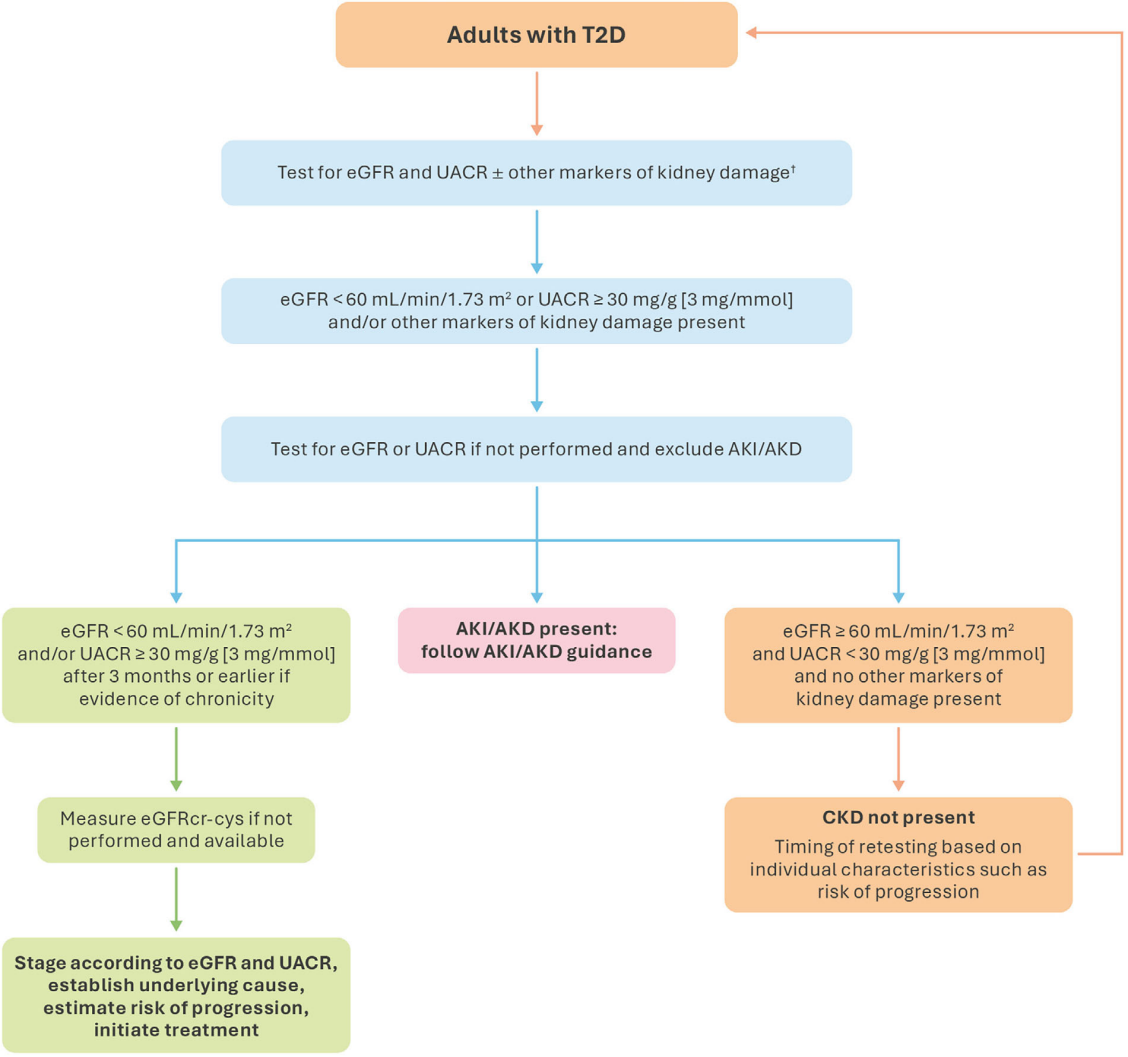
Screening algorithm for diagnosis and staging of CKD in adults. ^†^Markers of kidney damage other than albuminuria may also be used to diagnose CKD, but UACR and eGFR are still required to determine stage and estimate risk of progression. The orange boxes indicate actions in people at risk for CKD and in whom testing should be performed. The blue boxes indicate testing steps. The green boxes indicate the identification of CKD and its stages and the initiation of treatment. The pink box indicates the identification of AKD/AKI. AKD, acute kidney disease; AKI, acute kidney injury; CKD, chronic kidney disease; eGFRcr‐cys, creatinine and cystatin C–based estimated glomerular filtration rate; eGFR, estimated glomerular filtration rate; T2D, Type 2 diabetes; UACR, urine albumin‐to‐creatinine ratio. Source: Adapted from KDIGO 2024 [4] under the terms of the CC BY‐NC‐ND license.

Both eGFR and UACR testing are recommended when screening for CKD in people with T2D (Fig. 3) [4]. UACR is a sensitive and early indicator of kidney damage and can be routinely used to accurately assess the severity of CKD and monitor kidney health [36]. UACR values of ≥30 mg/g (≥3 mg/mmol) that persist for 3 months, in the absence of other indicators or markers of kidney damage, are alone sufficient to diagnose CKD and to prescribe therapy for slowing the progression of CKD [4, 37]. These recommendations are based on the findings of clinical trials that demonstrated improved outcomes with these therapies when albuminuria inclusion criteria were applied independently of other laboratory values [31, 38, 39].

Routine assessment of UACR can help potentially identify CKD before eGFR falls below 60 mL/min/1.73 m^2^ and before the onset of symptoms associated with advanced disease [34]. In a retrospective study of participants with normal‐range eGFR, mildly elevated UACR (≥7.0 mg/g) was associated with an increased incidence of CKD, a finding that suggests that UACR may be able to predict CKD early, prior to a decline in eGFR [40].

The American Diabetes Association (ADA) 2024 guidelines recommend, at a minimum, annual UACR and eGFR assessments in all individuals with T2D, regardless of treatment [37]. In people with diabetes and existing CKD with a higher risk of progression of kidney disease, UACR and eGFR should be monitored more frequently to guide treatment decisions [37].

The ADA and KDIGO recommend annual screening of individuals with diabetes for CKD [4, 37], and the KDIGO codified CKD classification based on eGFR and albuminuria is endorsed by the ADA and the ESC (Fig. 1). The KDIGO heatmap is a tool that can be used to diagnose CKD and when assessing the risk associated with CKD severity in individuals, with eGFR mapped on the left and albuminuria staging across the top. The color‐coded heatmap helps with easy identification of different levels of risk for CKD progression, acute kidney injury, CVD, CVD mortality, and all‐cause mortality [4, 7, 41, 42].

The US Kidney Disease Outcomes Quality Initiative and the ESC also recommend UACR and eGFR assessments for individuals with diabetes [43, 44]. Furthermore, the ESC guidelines recommend identifying CVD risk in individuals by the presence of severe target‐organ damage assessed by reduced eGFR and elevated UACR [44]. It is recommended that individuals with diabetes are routinely screened for kidney disease by assessing eGFR (defined by the CKD Epidemiology Collaboration equations) and UACR [44].

The American Heart Association guidelines on CV–kidney–metabolic health recommend that among adults with CV–kidney–metabolic disease stage II (those with metabolic risk factors, moderate‐to‐high risk CKD, or both) or higher, UACR should be measured annually, in addition to eGFR estimation using serum creatinine or cystatin C to allow accurate KDIGO staging and CKD risk assessment [45]. More frequent screening is indicated for individuals with a higher KDIGO risk [45].

Historically, initial recommendations for urinary protein assessment included measurement of 24‐h total protein excretion; however, this is a time‐consuming procedure, which is subject to collection error. As a result, the spot collection of urine samples used to report UACR (in mg/g) is now recommended [37, 42, 44]. Individuals providing a sample should be instructed to avoid intense exercise for 24 h before UACR sampling, and samples should not be collected if the person is experiencing fever or infection [46]. When compared with other available measures of albuminuria (e.g., 24‐h urinary albumin excretion and urinary albumin concentration), UACR demonstrates the highest accuracy for predicting kidney events in individuals with kidney disease and T2D [47].

Albuminuria remains a key diagnostic tool, with increases in albuminuria being associated with the early structural changes observed on kidney biopsy and poorer outcomes. Changes in albuminuria can also be a prognostic indicator of kidney disease progression or regression [36]. Inclusion of UACR in the Predicting Risk of CVD Events equations significantly improved model discrimination in terms of CV events in people with marked albuminuria [48]. There is also supporting evidence suggesting that UACR can predict incident hospitalization for HF in persons with CKD; analysis from the Chronic Renal Insufficiency Cohort Study demonstrated that the addition of UACR to the Pooled Cohort Equations to Prevent Heart Failure model improved the model performance and was able to better identify at‐risk individuals [49].

Regular monitoring of UACR in‐line with guideline recommendations is an essential tool in the detection of the onset of CKD, monitoring of disease progression, collection of kidney and CV prognostic data, and guidance in treatment decision‐making. However, rates of UACR testing remain suboptimal despite the recommendations for eGFR and UACR screening. United States and international cohort studies in participants with diabetes and/or hypertension have demonstrated that although eGFR testing rates are consistently high at approximately 90%, UACR testing rates are low. These studies indicate that approximately half to two‐thirds of participants with diabetes have not been screened for UACR, and testing for UACR is particularly low in persons with hypertension (≤11%) leaving many individuals at risk of CKD with undetected albuminuria due to lack of testing [16, 17, 18, 19].

Poor adherence to guideline recommendations on UACR testing may lead to a delay in diagnosis of CKD and the initiation of treatment resulting in poorer clinical outcomes and increased economic burden [50]. In people with T2D, a UACR of 300 mg/g is associated with significantly higher healthcare utilization and costs compared with a UACR <30 mg/g, including an almost threefold increase in all‐cause hospital admissions and fourfold increase in inpatient days [51]. A retrospective cohort study of people with CKD and T2D demonstrated that UACR testing reduced all‐cause mortality by 8% [52]. UACR testing in the early stages of CKD is cost‐effective in people with T2D, as the cost of testing and increased management is offset by fewer CV deaths, a reduced need for dialysis, and the generation of additional life‐years gained before end‐stage kidney disease [53].

The low testing rate of UACR compared with eGFR may reflect healthcare‐provider perceptions on the relative importance of the two tests in detecting and monitoring CKD, which may be influenced by the requirement for eGFR testing for guiding drug dosing with many CKD medications [19]. A lack of awareness by primary‐care providers of the prevalence of albuminuria in individuals with diabetes and hypertension, and its effect on CKD progression may be an important barrier to the uptake of UACR screening, and this may be compounded by low awareness of CKD among persons with diabetes and hypertension and limited time and resources [17]. Although serum creatinine for evaluation of eGFR may be incorporated into routine blood testing, there may be some hesitation regarding screening for UACR because of the additional urine testing required [19].

A lack of concise CKD guidelines may also contribute to low adherence to recommendations on UACR screening. The higher rate of UACR testing in individuals with diabetes compared with hypertension may reflect the more established, consistent guideline recommendations on testing for individuals with diabetes, as well as the availability of national performance data, which promotes annual assessments in this population [16, 18].

In high‐income countries, serum creatinine and eGFR are usually available during blood tests. Research into UACR‐based screening methods to assess early CKD at the population level is ongoing. Home‐based UACR screening was studied in 4484 individuals from the Netherlands between November 2019 and March 2021 3.3% of participants had an increased UACR [54]. There were 124 participants with a confirmed case of increased UACR via home‐based screening who underwent elaborate screening for CKD and CVD risk factors at hospital; 54.8% of these individuals were found to have one or more known risk factors outside the target range [54]. Physician support, the introduction of systematic workflow approaches within medical practices to ensure timely UACR testing for people with diabetes, and remote UACR monitoring may be useful for improving screening rates [50, 51, 55]. In addition, as primary‐care physicians are less likely to correctly identify people with advanced CKD than nephrologists, more education and collaboration between primary‐care physicians and specialists are required [56].

### Treatment recommendations to reduce UACR for persons with CKD

**Fig. 4:**
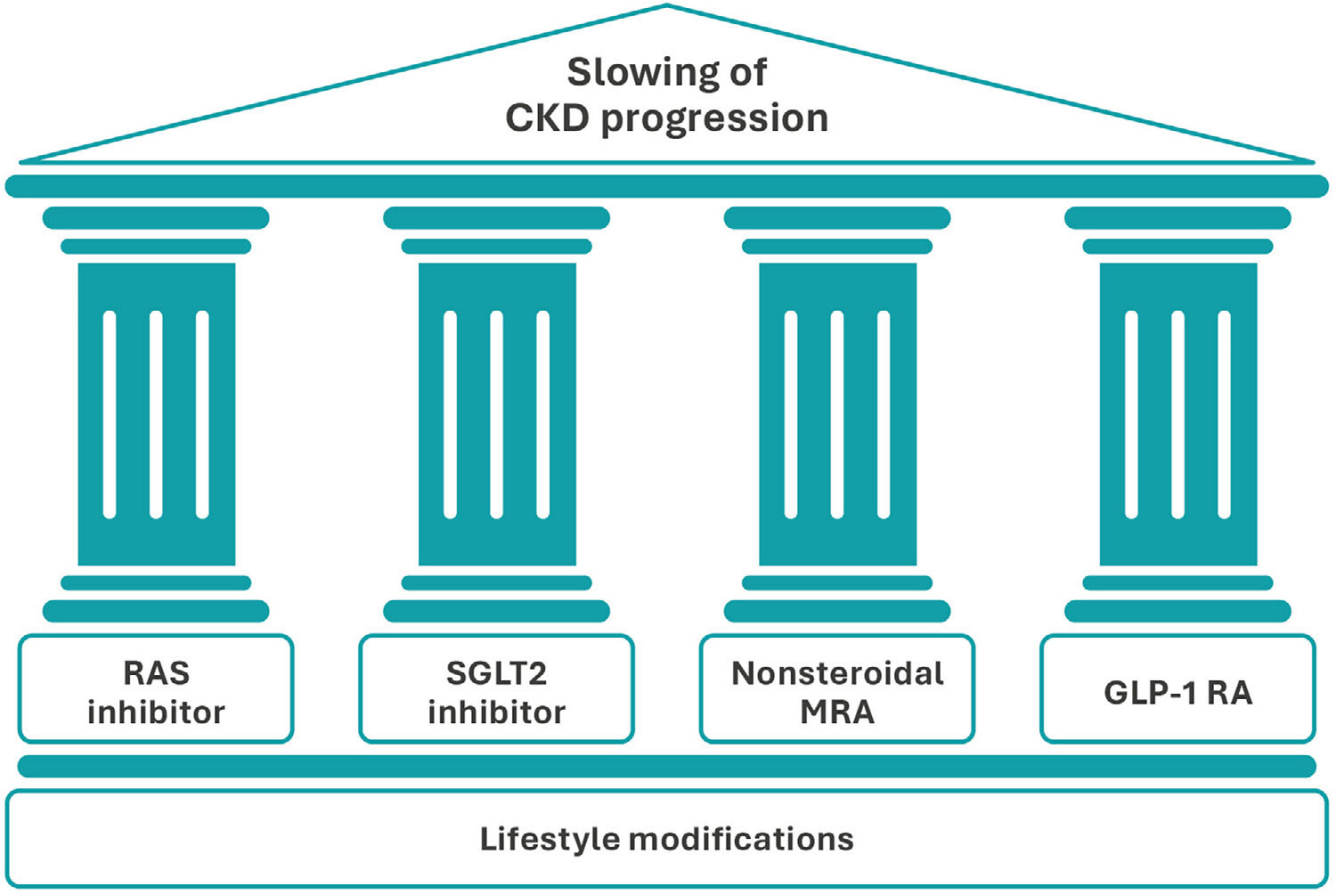
Pillars of therapy for slowing CKD progression in T2D. CKD, chronic kidney disease; GLP‐1 RA, glucagon‐like peptide 1 receptor agonist; MRA, mineralocorticoid receptor antagonist; RAS, renin–angiotensin–system; SLGT2, sodium–glucose cotransporter 2; T2D, Type 2 diabetes.

The treatment for slowing diabetic kidney disease has evolved over the past 50 years to a four‐pillar approach based on a strong evidence base and guideline recommendations [57, 58]. The four treatment approaches are the use of renin–angiotensin system (RAS) inhibition (angiotensin‐converting enzyme inhibitors [ACEis] or angiotensin receptor blockers [ARBs]), sodium–glucose cotransporter 2 (SGLT2) inhibitors, glucagon‐like peptide 1 receptor agonists (GLP‐1 RAs), and the nonsteroidal mineralocorticoid receptor antagonist (MRA) finerenone (Fig. 4) [58]. When used in combination, evidence indicates that these agents provide additive cardiorenal RR compared with RAS inhibitors alone [57]. In addition, lifestyle changes and intensive management of lipid abnormalities with a statin‐based regimen are recommended to reduce the risk of CVD in individuals with CKD and diabetes [44].

### RAS inhibitors

The RAS is central to blood pressure (BP) regulation, and fluid and electrolyte balance [59]. RAS inhibition with ACEis or ARBs reduces the activity of angiotensin, suppressing vasoconstriction and reducing BP [60]. Lowering BP is part of the foundation for slowing the progression of CKD and reducing the risk of CVD [61]. ACEis and ARBs are effective antihypertensive agents, and they have been the mainstay treatment for CKD for the last 20 years, being recommended as first‐line therapy due to their BP‐lowering and antiproteinuric effects [61, 62]. Both ACEis and ARBs mediate antiproteinuric effects via a reduction in glomerular hypertension through efferent arteriolar vasodilation. In addition, ACEis improve metabolic control, which attenuates any structural changes to the glomerulus [63].

A meta‐analysis of 119 trials of 64,768 participants with CKD reported that the use of ACEis or ARBs, when compared with placebo, reduced major CV events (odds ratio [95% credible interval] 0.82 [0.71–0.92] and 0.76 [0.62–0.89], respectively); however, they did not cause a significant reduction in CV death [64]. Evidence supporting CV benefit from RAS inhibition in individuals with CKD in diabetes is limited owing to the lack of large studies directly analyzing this population, and further research is required. However, given the association between CKD progression and CVD, event rates for kidney outcomes in participants with CKD and T2D randomized to placebo in addition to maximally tolerated/clinically appropriate RAS blockade in contemporary SGLT2 inhibitor trials provide insight on expected outcomes with ACEis and ARBs [65]. Event rates per 1000 patient‐years for kidney disease progression and kidney failure were 41–60 and 29–39, respectively [65]. In placebo‐treated participants on maximally tolerated RAS blockade in the FIDELITY study, a pooled analysis of Phase 3 trials with finerenone, event rates per 1000 patient‐years for kidney disease progression (using an eGFR ≥57% composite kidney outcome) and kidney failure were 25.5 and 16.2, respectively [66].

The main adverse effect of RAS inhibitor therapy is hyperkalemia, particularly in the advanced stages of disease [67]. A systematic review and meta‐analysis of 20 studies covering 47,122 participants reported that hyperkalemia is twice as common (risk ratio 2.03; 95% CI 1.67–2.48) in participants on drugs that act on the RAS compared with the control group [68]. In most individuals, the clinical benefit of RAS inhibition outweighs the risk of hyperkalemia [69]. Moreover, the risk can be managed without the need to discontinue therapy with RAS inhibitors by using strategies such as reducing potassium intake or prescribing loop diuretics and potassium binders [69], and the risk of hyperkalemia is lower in people on SGLT2 inhibitors [70].

### SGLT2 inhibitors

SGLT2 inhibitors reduce glucose and sodium reabsorption by kidney proximal tubular cells, by their action on SGLT2, a sodium‐coupled glucose transporter, which is upregulated in diabetes [71].

Data from randomized controlled trials (CREDENCE, SCORED, DAPA‐CKD, and EMPA‐KIDNEY) demonstrate improvements in cardiorenal outcomes following SGLT2‐inhibitor treatment in diabetic kidney disease (and non‐diabetic CKD) [72, 73, 74, 75, 76, 77].

CREDENCE (NCT02065791) was a double‐blind, randomized trial evaluating the effects of canagliflozin on kidney and CV outcomes in participants with T2D and albuminuric CKD who were randomized to receive canagliflozin or placebo in addition to maximally tolerated/clinically appropriate RAS blockade [72]. The primary endpoint was a composite kidney outcome, including end‐stage kidney disease (dialysis, transplantation, or a sustained eGFR of <15 mL/min/1.73 m^2^), a doubling of the serum creatinine level, or death from kidney or CV causes. In participants receiving canagliflozin, the relative risk of the composite kidney outcome was 30% lower compared with the placebo group, with event rates of 43.2 and 61.2 per 1000 patient‐years, respectively (HR 0.70; 95% CI 0.59–0.82). The risk of CV death, MI, or stroke was also lowered (HR 0.80; 95% CI 0.67–0.95). In participants with a history of CVD, the relative risk of the composite kidney outcome was 30% lower compared with the placebo group (HR 0.70; 95% CI 0.56–0.88). The event rates per 1000 patient‐years were 46.8 and 65.1 in the canagliflozin and placebo arm, respectively [72].

A secondary analysis of the CREDENCE trial investigated whether the effects of canagliflozin on clinically important kidney, CV, and safety outcomes were consistent across different ranges of eGFR [73]. The effect of canagliflozin on the primary composite outcome (end‐stage kidney disease, doubling of serum creatinine, or death from kidney or CV causes) was consistent in all eGFR categories. Of note, the composite outcome of CV death or hospitalization for HF was reduced in participants with screening eGFR of 30 to <45 mL/min/1.73 m^2^ (HR 0.69; 95% CI 0.50–0.94) [73]. An additional subgroup analysis evaluating the association between baseline UACR and the effects of canagliflozin demonstrated that higher UACR was associated with higher rates of kidney and CV events. Canagliflozin reduced efficacy outcomes irrespective of UACR level; kidney disease progression or CV death was reduced by 24% (HR 0.76; 95% CI 0.56–1.04) in the lowest UACR subgroup and by 37% (HR 0.63; 95% CI 0.47–0.84) in the highest subgroup (*p*_heterogeneity_ = 0.55) [74].

SCORED (NCT03315143) was a double‐blind, randomized, placebo‐controlled trial that compared sotagliflozin with placebo in addition to standard care in participants with T2D and CKD (>88% of participants received an RAS inhibitor) [75]. The primary endpoint (composite of the total number of deaths from CV causes, hospitalizations for HF, and urgent visits for HF) was reduced by 26% in participants receiving sotagliflozin compared with placebo (HR 0.74; 95% CI 0.63–0.88; *p *< 0.001). The rates of primary endpoint events were 5.6 and 7.5 events per 100 patient‐years in the sotagliflozin and placebo groups, respectively [75]. In participants with a history of CVD, the relative risk of the composite primary endpoint was 25% lower compared with the placebo group (HR 0.75; 95% CI 0.62–0.92).

DAPA‐CKD (NCT03036150) was a double‐blind, placebo‐controlled, randomized study evaluating the effect of dapagliflozin in participants with CKD, with or without T2D [76]. Participants were randomized to receive dapagliflozin or placebo in addition to stable RAS therapy. The primary outcome was a composite of a sustained decline in the eGFR of at least 50%, end‐stage kidney disease, or death from kidney or CV causes. The secondary CV composite outcome was a composite of death from CV causes or hospitalization for HF. The relative risk of the primary composite outcome was 39% lower in the dapagliflozin arm compared with the placebo group, with event rates of 4.6 and 7.5 per 100 patient‐years, respectively (HR 0.61; 95% CI 0.51–0.72). The relative RR for the primary composite outcome with dapagliflozin was consistent in participants with T2D (HR 0.64; 95% CI 0.52–0.79) and those without T2D (HR 0.50; 95% CI 0.35–0.72). The secondary CV composite outcome was reduced in the dapagliflozin arm by 29% (HR 0.71; 95% CI 0.55–0.92).

A prespecified subgroup analysis of the DAPA‐CKD trial evaluating the effects of dapagliflozin according to history of CVD demonstrated that the risk of the primary composite outcome was higher in participants with a history of CVD, with an event rate of 7.0 per 100 person‐years compared with an event rate of 5.4 per 100 person‐years in participants without a history of CVD [78]. The treatment effect of dapagliflozin on reducing the primary composite outcome was similar in participants with a history of CVD (HR 0.61; 95% CI 0.47–0.79) or without (HR 0.61; 95% CI 0.48–0.78; *p*_interaction_ = 0.90). Another prespecified analysis of the DAPA‐CKD trial investigating the effects of dapagliflozin according to the number of baseline diabetes medications demonstrated that the effect of dapagliflozin on the primary composite endpoint was consistent across groups (HR 0.64; 95% CI 0.52–0.79) [79].

In the EMPA‐KIDNEY trial (NCT03594110), empagliflozin reduced the primary outcome of progression of kidney disease or death from CV causes in participants with diabetes compared with placebo (HR 0.64; 95% CI 0.54–0.77) [77]. The secondary outcome of hospitalization for HF or death from CV causes was also reduced in the empagliflozin group compared with placebo (HR 0.84; 95% CI 0.67–1.07). Empagliflozin reduced progression of kidney disease or death from CV causes by 27% (HR 0.73; 95% CI 0.58–0.92) in the subgroup of participants with CVD at baseline, and the treatment effect of empagliflozin was generally consistent across all other prespecified subgroups [77].

A systematic review and meta‐analysis of these four CKD outcomes trials reported that SGLT2 inhibitor use reduced kidney disease progression by 38% (RR 0.62; 95% CI 0.56–0.69) with similar RRs in participants with and without diabetes [65]. The relative RR in kidney disease progression with SGLT2 inhibitors across the four trials was similar when analyzed separately by primary kidney diagnosis (diabetic kidney disease or nephropathy, ischemic and hypertensive kidney disease, glomerular disease, and other/unknown). Across the four trials, SGLT2 inhibitors reduced CVD death or hospitalization for HF in participants with diabetes (RR 0.74; 95% CI 0.66–0.82) but not in individuals without diabetes (RR 0.95; 95% CI 0.65–1.40).

Post hoc analyses of randomized controlled trials of SGLT2 inhibitors in participants with T2D and high CVD risk have demonstrated improvements in cardiorenal outcomes irrespective of baseline KDIGO risk category (i.e., including participants who did not have CKD at baseline, as diagnosed by eGFR or UACR criteria) [80, 81, 82]. In the CANVAS program, the reduction in the primary outcome (CV death, nonfatal MI, or nonfatal stroke) with canagliflozin (HR 0.86; 95% CI 0.75–0.97) was consistent across KDIGO risk categories (*p*_trend_ = 0.2). Similar findings were reported for other CV and kidney outcomes [80]. Dapagliflozin reduced cardiorenal and kidney‐specific composite outcomes across all KDIGO risk categories in DECLARE‐TIMI 58 (*p*_interaction_ = 0.151 and 0.968, respectively). In the low KDIGO risk category (i.e., no CKD present), the kidney‐specific outcome was reduced by 46% compared with placebo (HR 0.54; 95% CI 0.38–0.77) [81]. In EMPA‐REG OUTCOMES, RRs with empagliflozin were consistent across KDIGO categories for CV outcomes (*p*_interaction_ range = 0.26–0.85) and kidney outcomes (*p*_interaction_ range = 0.16–0.60) [82]. The findings of these studies demonstrate that SGLT2 inhibitors reduce cardiorenal risk even in participants in low KDIGO risk categories, suggesting that SGLT2 inhibitors may have benefits in the prevention of diabetic kidney disease in people who do not have CKD at baseline.

### GLP‐1 RAs

GLP‐1 RAs increase hyperglycemia‐induced insulin secretion and were developed to improve glycemic control in individuals with T2D [83]. They slow gastric emptying and suppress appetite and promote weight loss and improve CVD risk factors in persons with obesity [84]. GLP‐1 RAs demonstrate CV benefits in individuals with T2D [83]. They have also been shown to reduce the rate of albumin excretion in persons with albuminuria and T2D [58]. Although GLP‐1 RAs have a well‐characterized safety profile, the drug class has been associated with mild gastrointestinal adverse reactions [83].

A meta‐analysis of eight outcomes trials in 60,080 participants with T2D showed that GLP‐1 RAs reduced the composite kidney outcome (macroalbuminuria, doubling of serum creatinine, ≥40% decline in eGFR, kidney replacement therapy or death from kidney failure, or worsening of kidney function) by 21% (HR 0.79; 95% CI 0.73–0.87). The same analysis reported a 14% reduction in major adverse CV events after treatment with GLP‐1 RAs (HR 0.86; 95% CI 0.80–0.93) [85].

A dedicated kidney outcomes trial (FLOW; NCT03819153) assessed semaglutide compared with placebo in participants with T2D and CKD [86]. The trial was terminated following a prespecified interim analysis because efficacy criteria had been met. The primary endpoint (composite of the onset of kidney failure, at least a 50% reduction in eGFR from baseline, or death from kidney‐related or CV causes) was reduced by 24% (HR 0.76; 95% CI 0.66–0.88) with semaglutide versus placebo. Semaglutide reduced death from CV causes by 29% (HR 0.71; 95% CI 0.56–0.89) and the risk of major CV events by 18% (HR 0.82; 95% CI 0.68–0.98) compared with placebo.

### MRAs

In the kidneys, mineralocorticoid receptors (MRs) are responsible for regulating fluid and electrolyte homeostasis. The overactivation of MRs in the kidneys may contribute to fibrosis, decline in eGFR, proteinuria, progressive kidney function loss, and inflammation [87, 88].

There are two different classes of MRAs: steroidal and nonsteroidal.

### Steroidal MRAs

Over the past decades, two MRAs with steroidal chemical structures were developed: spironolactone and eplerenone. Spironolactone and eplerenone are approved for increasing survival in persons with HF with reduced ejection fraction [89, 90] based on results from landmark trials [91, 92]. Studies of spironolactone and eplerenone in participants with HF with preserved ejection fraction have demonstrated a lack of efficacy in this setting [93, 94, 95]. Consequently, neither have an indication in HF with preserved ejection fraction [90, 96].

Despite hyperkalemia being relatively uncommon and not depriving participants with HF of survival benefit in clinical trials of spironolactone and eplerenone, data from real‐world studies have shown a high incidence of hyperkalemic events in individuals treated with these therapies [97, 98]. The higher rates of hyperkalemia with steroidal MRAs in observational studies compared with clinical trials may be associated with inappropriate use of high doses, use in persons with conditions that predisposed them to hyperkalemia, and limited serum potassium monitoring [99, 100, 101]. In addition, spironolactone (but not eplerenone) is associated with increased gynecomastia [93].

Data from HF outcomes trials with spironolactone and eplerenone indicated that they did not delay the progression of CKD [102, 103], even in high‐risk participants [104]. Due to the lack of long‐term clinical trial data in individuals with CKD, neither spironolactone nor eplerenone is indicated for use in this population. Thus, nonsteroidal MRAs were developed to improve long‐term clinical outcomes in persons with CKD.

### Nonsteroidal MRAs

Nonsteroidal MRAs were developed with the aim of improving the adverse event profile compared with steroidal MRAs while maintaining a potent blockade of the MR receptor. Only one nonsteroidal MRA, finerenone, is approved for slowing CKD progression in individuals with CKD in diabetes [105, 106]. Due to its unique binding mechanism, finerenone demonstrates increased selectivity for the MR over spironolactone and eplerenone [88, 107]. Finerenone also has a shorter half‐life compared with spironolactone and eplerenone [88, 107] and recruits a different set of transcriptional coactivators [108].

Two studies have examined the effect of finerenone on CV and kidney outcomes in participants with T2D and CKD characterized by UACR ≥30 mg/g: the FIDELIO‐DKD and the FIGARO‐DKD studies [109, 110]. In the FIDELIO‐DKD trial, finerenone reduced the occurrence of the primary composite kidney endpoint (kidney failure, a sustained decrease of at least 40% in the eGFR from baseline, or death from kidney causes) by 18% compared with placebo (HR 0.82; 95% CI 0.73–0.93) [110]. In FIGARO‐DKD, finerenone reduced the occurrence of the primary CV endpoint (composite of death from CV causes, nonfatal MI, nonfatal stroke, or hospitalization for HF) by 13% compared with placebo (HR 0.87; 95% CI 0.76–0.98) [109]. Furthermore, the incidence of new‐onset HF was significantly lower with finerenone than with placebo (HR 0.68; 95% CI 0.50–0.93) [111].

Pooled data from FIDELIO‐DKD and FIGARO‐DKD were analyzed in the prespecified FIDELITY study, which demonstrated the efficacy of finerenone in improving CV and kidney failure outcomes in a broad spectrum of persons with T2D and CKD [66]. The risk of the primary CV composite outcome was reduced by 14% in participants treated with finerenone compared with placebo (HR 0.86; 95% CI 0.78–0.95). Hospitalization for HF was also significantly reduced in participants treated with finerenone compared with placebo (HR 0.78; 95% CI 0.66–0.92). The composite kidney outcome was reduced by 23% with finerenone compared with placebo (HR 0.77; 95% CI 0.67–0.88).

Subgroup analyses of FIDELITY demonstrated that neither SGLT2 inhibitor nor GLP‐1 RA use at baseline affected the RR in the primary CV composite outcome with finerenone [112, 113]. The HRs (95% CIs) were 0.67 (0.42–1.07) and 0.87 (0.79–0.96) in participants treated with and without an SGLT2 inhibitor, respectively [112]. Finerenone reduced the risk of the CV composite outcome by 24% (HR 0.76; 95% CI 0.52–1.11) in participants who were treated with GLP‐1 RAs at baseline compared with 13% (HR 0.87; 95% CI 0.79–0.96) in participants without GLP‐1 RA use at baseline [113].

In FIDELITY, hyperkalemia‐related adverse events were more frequent in the finerenone group versus the placebo group (14% vs. 6.9%, respectively); however, none were fatal and only a small proportion resulted in permanent treatment discontinuation (1.7% [incidence rate 0.66 per 100 patient‐years] and 0.6% [incidence rate 0.22 per 100 patient‐years], respectively) or hospitalization (0.9% and 0.2%, respectively) [66].

### Guideline recommendations

A joint group of ADA and KDIGO representatives developed a series of consensus statements to identify and highlight shared recommendations from the ADA 2022 Standards of Medical Care in Diabetes and KDIGO 2022 Clinical Practice Guideline for Diabetes Management in CKD [42]. The consensus report found that the published guidelines are aligned in the areas of CKD screening and diagnosis, glycemia monitoring, lifestyle therapies, treatment goals, and pharmacologic management. Both the KDIGO and ADA recommend the use of ACEis and ARBs at the maximum‐tolerated dose in persons with diabetes who have hypertension and albuminuria [37, 42]. The recommendation is based on the results from randomized controlled trials that have demonstrated a decreased risk of CKD progression in persons treated with RAS inhibitors compared with a placebo or an active antihypertensive drug comparator. The consensus guidelines also recommend the use of an SGLT2 inhibitor with proven kidney or CV benefit for persons with T2D, CKD, and eGFR ≥20 mL/min/1.73 m^2^. Use of an SGLT2 inhibitor can be continued at lower levels of eGFR per the treatment guidelines [42]. The use of a GLP‐1 RA with proven CV benefit is recommended for persons with T2D and CKD who do not meet their individualized glycemic target with metformin alone, in combination with an SGLT2 inhibitor or an SGLT2 inhibitor alone. GLP‐1 RAs are also recommended for persons who are unable to use these drugs [4, 42]. The aims of GLP‐1 RA prescription are likely to be updated based on the results of the FLOW study [86]. A nonsteroidal MRA with proven kidney and CV benefit is recommended for persons with T2D, eGFR ≥25 mL/min/1.73 m^2^, normal serum potassium concentration, and albuminuria (UACR ≥30 mg/g) regardless of whether or not the individual is at the maximum tolerated dose of RAS inhibitor [4, 37, 42].

The ESC guidelines for treatment of persons with T2D and CKD recommend sequentially initiating and titrating doses of an ACEi or ARB, an SGLT2 inhibitor, finerenone, and a GLP‐1 RA in addition to BP control and a statin‐based regimen to reduce the risk of CVD and kidney failure [44]. The maximum tolerated dose of an ACEi or ARB is recommended. An SGLT2 inhibitor is recommended in persons with T2D and CKD with an eGFR ≥20 mL/min/1.73 m^2^. Finerenone is recommended in addition to an ACEi or ARB in persons with T2D and eGFR >60 mL/min/1.73 m^2^ with a UACR ≥300 mg/g, or eGFR 25–60 mL/min/1.73 m^2^ and UACR ≥30 mg/g [44]. A GLP‐1 RA is recommended at eGFR >15 mL/min/1.73 m^2^.

### Pillar‐based approach and combination therapy

Based on data from key clinical trials evaluating the use of SGLT2 inhibitors, GLP‐1 RAs, and the nonsteroidal MRA finerenone in addition to background RAS therapy in participants with CKD and diabetes, a pillared approach to therapy has been suggested, which is similar to the pillared approach that is currently in use within the treatment landscape for HF [57, 58]. Lifestyle modifications are the foundation on which the pillars are built (Fig. 4). These include, but are not limited to, cessation of smoking, glycemic control, BP lowering, management of dyslipidemia, dietary modification, including restricting sodium intake, exercise, and maintaining a healthy weight [58, 71].

The increasing body of evidence supporting the additive cardiorenal RR when different therapeutic options are used in combination with a background of maximally dosed RAS blockers has led to the recommendation of combination therapy [57, 69]. Combination therapy with GLP‐1 RAs and SGLT2 inhibitors is recommended by the ADA and European Association for the Study of Diabetes 2018 consensus report for persons with T2D and atherosclerotic CVD and/or CKD who are not meeting their glycated hemoglobin targets with other medication [114]. The Diabetes, Cardiorenal, and Metabolic Diseases Task Force practice recommendations suggest, as part of a multispecialty consensus, adding finerenone in combination with an SGLT2 inhibitor to ARB or ACEi therapy for preventing HF and slowing CKD progression in persons with diabetes and CKD [115]. The combination of finerenone and glucose‐lowering medications in persons with T2D and persistent albuminuria was recommended in the latest ADA and KDIGO consensus statement [42].

## Conclusions

CKD in diabetes is a preventable and modifiable CVD risk factor. Early identification of CKD in T2D is important to slow progression and mitigate the risk of kidney failure and CV complications. UACR testing is an actionable component of the CKD definition. Spot UACR screening is a simple and effective strategy that can help with early identification and intervention to help prevent kidney failure and CV complications. Therefore, implementation and adherence to screening for UACR at the time of diagnosis of diabetes is critical; however, UACR screening is underused despite decades‐old guideline recommendations.

The pillars of CKD treatment in persons with T2D, RAS inhibition, SGLT2 inhibitors, finerenone, and GLP‐1 RAs, are built on the foundation of lifestyle modifications that include cessation of smoking, BP control, lipid management, physical activity, maintenance of a healthy weight, and a diet low in sodium. Several ongoing studies will provide more information on how the pillared approach to treatment could be incorporated into CKD treatment guidelines. These studies include the CONFIDENCE study (NCT05254002), which evaluates the effect of finerenone in combination with the SGLT2 inhibitor empagliflozin on the relative change in UACR from baseline in participants with T2D and CKD [116].

## Author contributions

**Faiez Zannad**: Conceptualization; visualization; supervision. **Darren K. McGuire**: Writing—review and editing. **Alberto Ortiz**: Conceptualization; writing—review and editing; visualization.

## Conflict of interest statement

Dr Zannad reports personal fees from 89Bio, Abbott, Acceleron, Applied Therapeutics, Bayer, Betagenon, Boehringer Ingelheim, BMS, CVRx, Cambrian, Cardior, Cereno pharmaceutical, Cell Prothera, CEVA, Inventiva, KBP, Merck, Novo Nordisk, Owkin, Otsuka, Roche Diagnostics, Northsea, USa2, having stock options at G3Pharmaceutical and equities at Cereno, Cardiorenal, Eshmoun Clinical Research, and being the founder of Cardiovascular Clinical Trialists.

Dr McGuire reports consulting fees from Boehringer Ingelheim, Lilly USA, Novo Nordisk, AstraZeneca, Lexicon Pharmaceuticals, Pfizer, Applied Therapeutics, Altimmune, Bayer, Neurotronics, Intercept Pharmaceuticals, Esperion, Ventyx Pharmaceuticals, New Amsterdam, CSL Behring, and Amgen.

Professor Ortiz has received grants from Sanofi and consultancy or speaker fees or travel support from Adviccene, Alexion, Astellas, AstraZeneca, Amicus, Amgen, Bioporto, Boehringer Ingelheim, Fresenius Medical Care, GSK, Bayer, Sanofi‐Genzyme, Sobi, Menarini, Mundipharma, Kyowa Kirin, Lilly, Freeline, Idorsia, Chiesi, Otsuka, Novo Nordisk, Sysmex and Vifor Fresenius Medical Care Renal Pharma and Spafarma and is Director of the Catedra UAM‐Mundipharma research collaboration for diabetic kidney disease and the Catedra UAM–AstraZeneca research collaboration for chronic kidney disease and electrolytes. He is a member of the European Renal Association Council and SOMANE. He has stock in Telara Farma.

## Funding information

This review was supported by Bayer AG. The authors wrote the paper independently with the assistance of a medical writer, who was funded by the sponsor. The sponsor is also the manufacturer of finerenone. AO's research is funded by Comunidad de Madrid en Biomedicina P2022/BMD‐7223, CIFRA_COR‐CM; the Instituto de Salud Carlos III (ISCIII) RICORS program to RICORS2040 (RD21/0005/0001, RD24/0004/0001) is co‐funded by the European Union; and COST Action PERMEDIK CA21165 is supported by COST (European Cooperation in Science and Technology). AO's research is also funded by the PREVENTCKD Consortium Project ID: 101101220 Programme: EU4H DG/Agency HADEA.

## Supporting information


Plain language summary
