# Supplementary material for: Treatment strategies to reduce cardiovascular risk in persons with chronic kidney disease and Type 2 diabetes
Source: J Intern Med. 2024 Dec 31;297(5):460–78. doi: 10.1111/joim.20050 (PMC12033002; doi:10.1111/joim.20050)
Supplement: Supplementary file 1 — Plain language summary [file JOIM-297-460-s001.docx]

# Supplementary materials

## Plain language summary

Chronic (long-term) kidney disease (CKD) is common. It is a disease that progressively worsens, and which may cause severe illness and death. The leading cause of death in individuals with CKD is cardiovascular disease (CVD), which includes heart attack and stroke. Diabetes is a common cause of CKD, and the risk of developing CVD is increased in individuals who have both diabetes and CKD.

CKD in individuals with diabetes is preventable and treatable. Early treatment is important to slow the progression of CKD and delay the onset of symptoms and other illnesses. People in the early stages of CKD often do not have symptoms, so they do not know that they have CKD. However, two laboratory tests can be used to assess how well the kidney is working; these tests measure kidney function and kidney injury. Kidney function is measured by the estimated glomerular filtration rate (eGFR) using a blood sample. Kidney injury is measured by the urine albumin-to-creatinine ratio (UACR) using urine. The results of both tests are required to make a diagnosis of CKD. Guidelines recommend that these tests should be performed at least annually in individuals with diabetes and in people with other conditions that put them at risk of developing CKD. eGFR is often tested in patients with diabetes, but UACR is underused, which may result in CKD remaining undiagnosed and not treated. Measuring UACR is important as it allows early diagnosis and treatment of CKD rather than waiting for the kidneys to (irreversibly) lose function.

On top of lifestyle changes, including improved diet and exercise, four types of treatment are recommended for slowing the rate at which CKD worsens in people with CKD and diabetes. These are renin–angiotensin–aldosterone system (RAAS) inhibitors, sodium–glucose cotransporter 2 inhibitors, glucagon-like peptide 1 receptor agonists, and the nonsteroidal mineralocorticoid receptor antagonist finerenone. These treatments are more effective at reducing the risk of CKD worsening and the development of CVD when they are used together compared with treatment with only RAAS inhibitors (the first of these treatments to be used). Early diagnosis of CKD and prompt treatment with these recommended therapies are crucial for reducing the number of people with CKD and diabetes that develop CVD or progress to kidney failure that requires dialysis. Additional research is ongoing that will further improve our understanding of how best to use these therapies for the treatment of CKD in people with diabetes.
